# Supplementary material for: Local ROS and inefficient autophagosome formation limit Optineurin-mediated mitophagy in neurons
Source: bioRxiv. 2026 Oct 3:2026.01.16.700014. Preprint. [Version 2] doi: 10.64898/2026.01.16.700014 (PMC13648377; doi:10.64898/2026.01.16.700014)
Supplement: Supplementary file 1 [file NIHPP2026.01.16.700014v2-supplement-1.pdf]

bioRxiv preprint doi: https://doi.org/10.64898/2026.01.16.700014; this version posted October 3, 2026. The copyright holder for this preprint (which was not certified by peer review) is the author/funder, who has granted bioRxiv a license to display the preprint in perpetuity. It is made available under a CC-BY 4.0 International license.

FIGURE S1

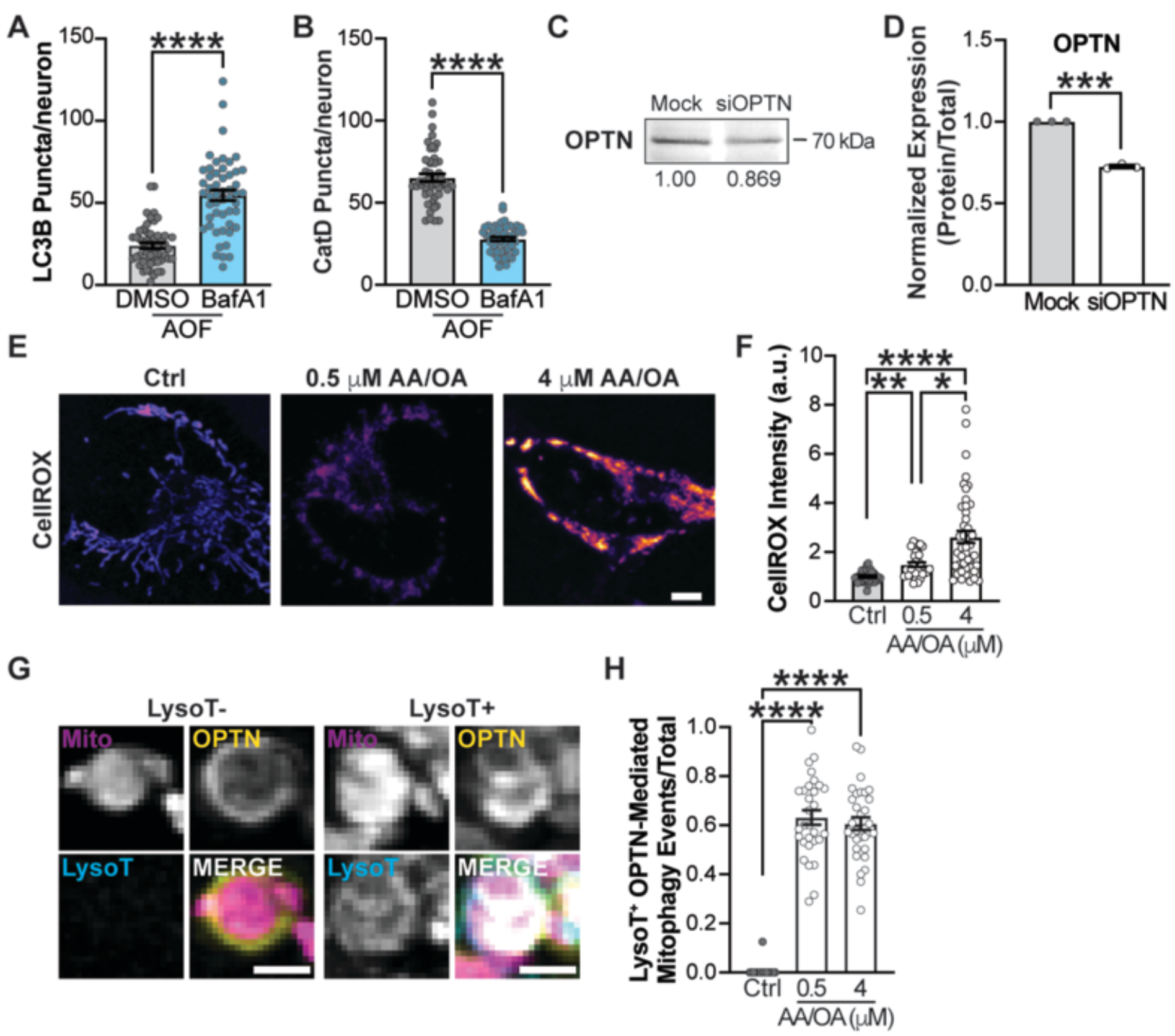

bioRxiv preprint doi: https://doi.org/10.64898/2026.01.16.700014; this version posted October 3, 2026. The copyright holder for this preprint (which was not certified by peer review) is the author/funder, who has granted bioRxiv a license to display the preprint in perpetuity. It is made available under a CC-BY 4.0 International license.

Figure S1: The levels of ROS do not alter the fast rates of mitochondrial turnover in HeLa cells. (**A**) Quantification of the number of LC3B+ puncta per neuron in the soma of hippocampal neurons following a 6-hour AOF treatment in either DMSO or BafA1. Mean ± SEM; n = 50-51 neurons from 5 biological replicates; 8-9 DIV. ****, $p < 0.0001$ by unpaired Mann-Whitney test. (**B**) Quantification of CatD+ puncta per neuron in the soma of hippocampal neurons following a 6-hour AOF treatment in either DMSO or BafA1. Mean ± SEM; n = 49-52 from 5 biological replicates; 8-9 DIV. ****, $p < 0.0001$ by unpaired Mann-Whitney test. (**C-D**) Representative fluorescent western blot (C) and quantification (D) of OPTN in neuron lysates. Data shown as the fold change over control of the protein of interest divided by the total protein stain. Normalization factors are shown under the corresponding representative images. Mean ± SEM; n = 3 biological replicates; 9 DIV. ***, $p < 0.001$ by Welch's test. (**E-F**) Representative images (E) and quantification (F) of intracellular ROS using CellROX Deep Red. HeLa cells were treated for 2 hours with either control, 0.5 µM AA/0.5 µM OA, or 4 µM AA/4 µM OA. Scale bar, 5 µm. Mean ± SEM; n = 33-46 cells from 3 biological replicates. *, $p < 0.05$; **, $p < 0.01$; ****, $p < 0.0001$ by Kruskal-Wallis with Dunn's multiple comparisons test. (**G-H**) Representative images (G) and quantification (H) of the fraction of OPTN-mediated mitophagy events that are LysoT+ following a 2-hour treatment. Scale bar, 1 µm. Mean ± SEM; n = 30-32 cells from 3 biological replicates. ****, $p < 0.0001$ by Kruskal-Wallis with Dunn's multiple comparisons test.

bioRxiv preprint doi: https://doi.org/10.64898/2026.01.16.700014; this version posted October 3, 2026. The copyright holder for this preprint (which was not certified by peer review) is the author/funder, who has granted bioRxiv a license to display the preprint in perpetuity. It is made available under a CC-BY 4.0 International license.

FIGURE S2

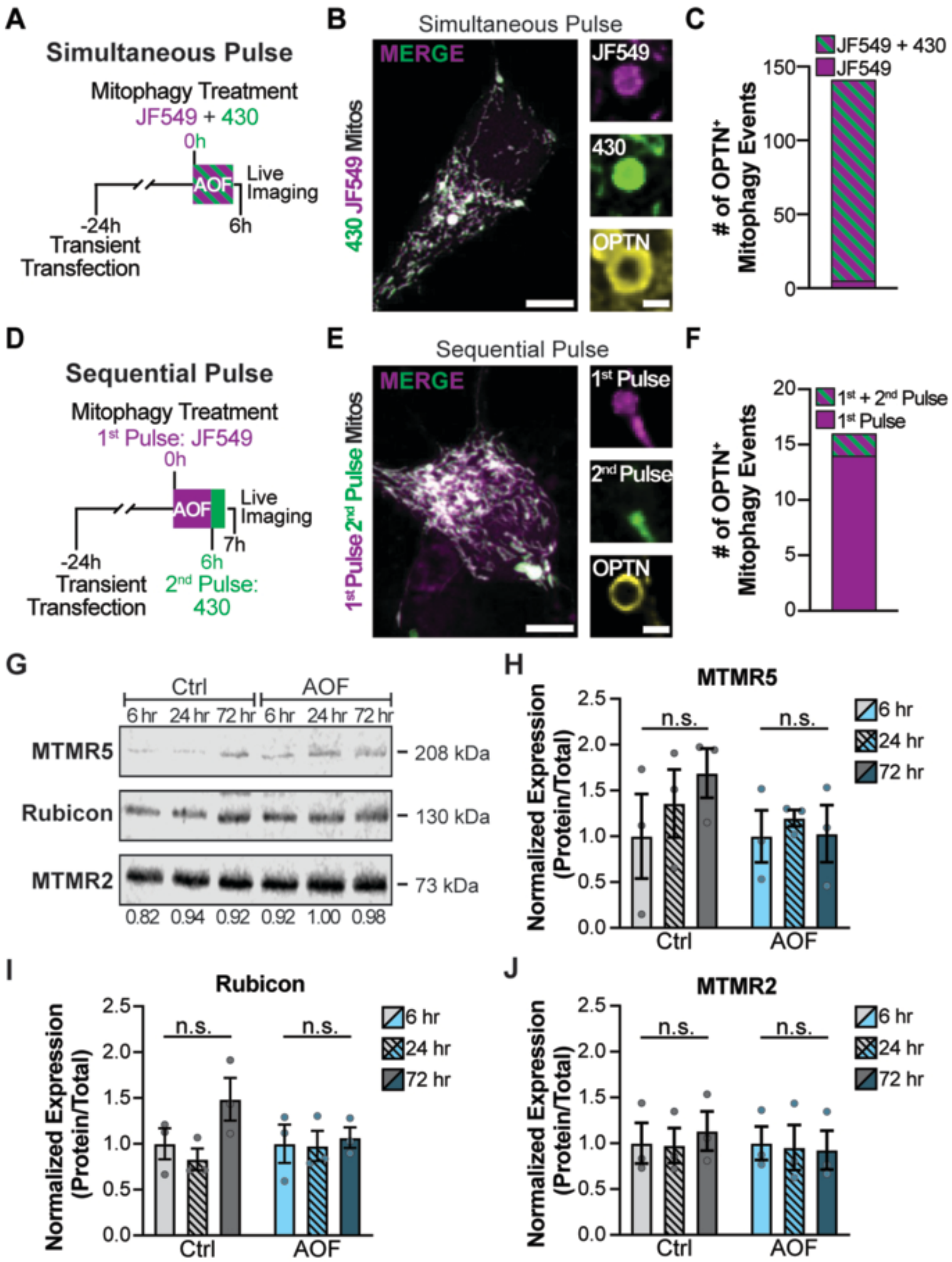

bioRxiv preprint doi: https://doi.org/10.64898/2026.01.16.700014; this version posted October 3, 2026. The copyright holder for this preprint (which was not certified by peer review) is the author/funder, who has granted bioRxiv a license to display the preprint in perpetuity. It is made available under a CC-BY 4.0 International license.

Figure S2: Negative autophagy regulators' abundance does not change throughout the pulse-chase paradigm. (**A**) Experimental timeline for simultaneous SNAP pulse-chase labeling with SNAP JF549 and SNAP 430. Briefly, transiently transfected neurons were treated with AOF for 6 hours to induce OPTN-dependent mitophagy and simultaneously labeled with two SNAP ligands. After the treatment ended, the neurons were immediately imaged. (**B**) Representative images of the somal mitochondrial network (left) and OPTN+ mitophagy event (right) following simultaneous SNAP labeling. Scale bar: 5 μm (left); 1 μm (right). (**C**) Quantification of the total number of OPTN+ mitophagy events that are single-labeled (JF549) or dual-labeled (SNAP JF549 + 430). *n* = 25 neurons from 3 biological replicates; 7 DIV. (**D**) Experimental timeline for sequential SNAP pulse-chase labeling. Briefly, transiently transfected neurons were treated in parallel with AOF for 6 hours and labeled with SNAP JF549. Next, neurons were immediately labeled with SNAP 430 for 1 hour and imaged. (**E**) Representative images of the mitochondrial network (left) and OPTN+ mitophagy event (right) following sequential SNAP pulses. Scale bar: 5 μm (left); 1 μm (right). (**F**) Quantification of the total number of OPTN+ mitophagy events labeled with the 1st Pulse (SNAP JF549) or 1st + 2nd Pulse (dual-labeled with SNAP JF549 and SNAP 430) when ligands were added sequentially. *n* = 31 neurons from 3 biological replicates; 7 DIV. (**G-J**) Representative fluorescent western blot (G) and quantification of MTMR5 (H), Rubicon (I), and MTMR2 (J) following 6-, 24-, or 72-hour recovery after control or AOF treatment. Data are shown as the fold change over the mean of the 6-hour recovery protein of interest divided by the total protein stain. Normalization factors are shown under the corresponding representative images. Mean ± SEM; *n* = 3 biological replicates; 7 DIV. n.s., not significant by One-way ANOVA with Tukey multiple comparisons test (H-I) and Brown-Forsythe and Welch's ANOVA with Dunnett T3 multiple comparisons (J).

bioRxiv preprint doi: https://doi.org/10.64898/2026.01.16.700014; this version posted October 3, 2026. The copyright holder for this preprint (which was not certified by peer review) is the author/funder, who has granted bioRxiv a license to display the preprint in perpetuity. It is made available under a CC-BY 4.0 International license.

FIGURE S3

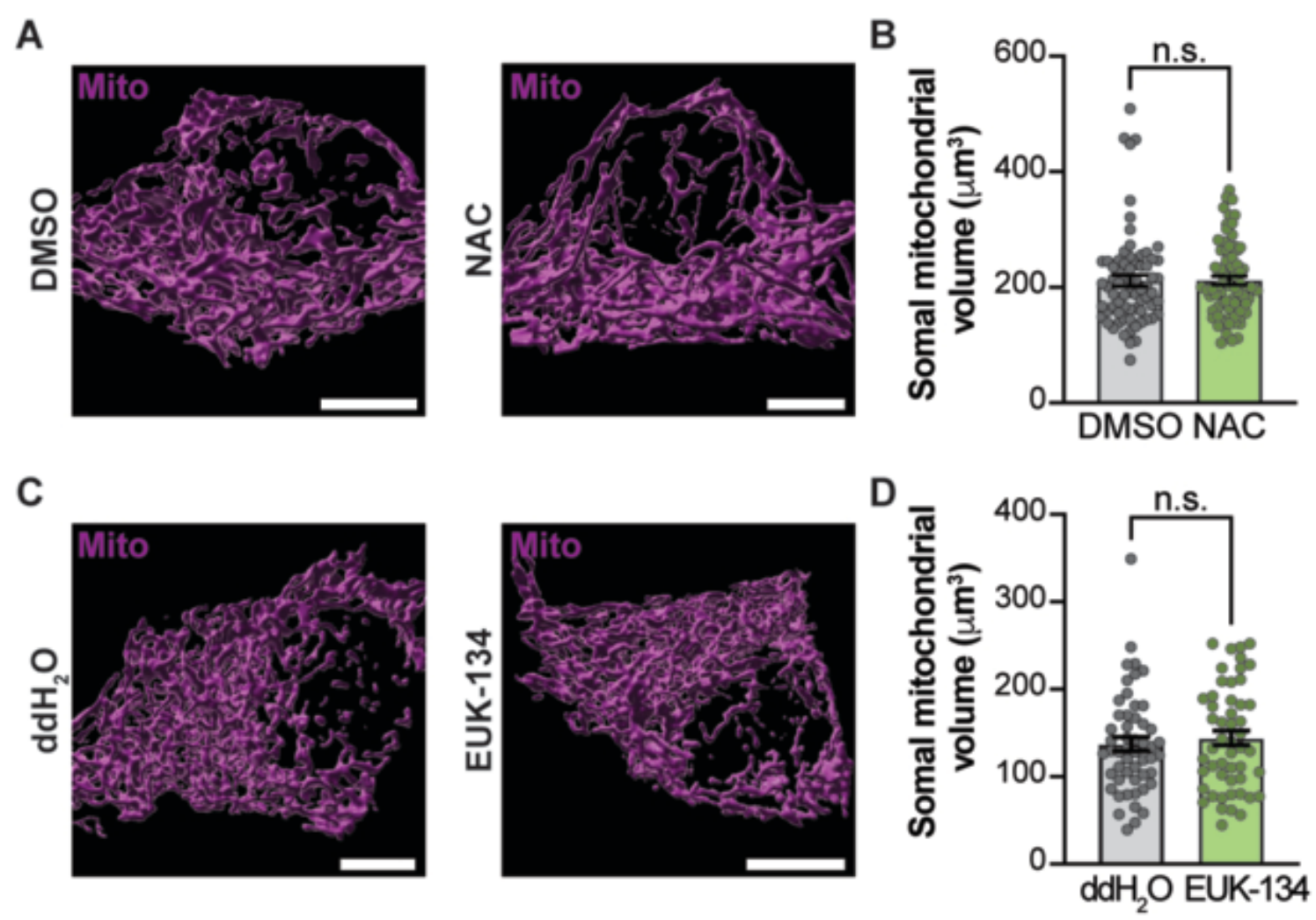

bioRxiv preprint doi: https://doi.org/10.64898/2026.01.16.700014; this version posted October 3, 2026. The copyright holder for this preprint (which was not certified by peer review) is the author/funder, who has granted bioRxiv a license to display the preprint in perpetuity. It is made available under a CC-BY 4.0 International license.

Figure S3. Treatment with NAC or EUK-134 does not alter mitochondrial volume. (**A-B**) Representative images (A) and quantification (B) of somal mitochondrial surface volumes in DMSO-treated (left) and NAC-treated (right) neurons. Scale bar, 5 μm. Mean ± SEM; *n* = 70 neurons from 4 biological replicates; 8 DIV. n.s., not significant by unpaired Mann-Whitney test. (**C-D**) Representative images (C) and quantification (D) of somal mitochondrial surface volumes in ddH_2O-treated (left) and EUK-134-treated (right) neurons. Scale bar, 5 μm. Mean ± SEM; *n* = 51-52 neurons from 3 biological replicates; 8 DIV. n.s., not significant by unpaired Mann-Whitney test.

bioRxiv preprint doi: https://doi.org/10.64898/2026.01.16.700014; this version posted October 3, 2026. The copyright holder for this preprint (which was not certified by peer review) is the author/funder, who has granted bioRxiv a license to display the preprint in perpetuity. It is made available under a CC-BY 4.0 International license.

FIGURE S4

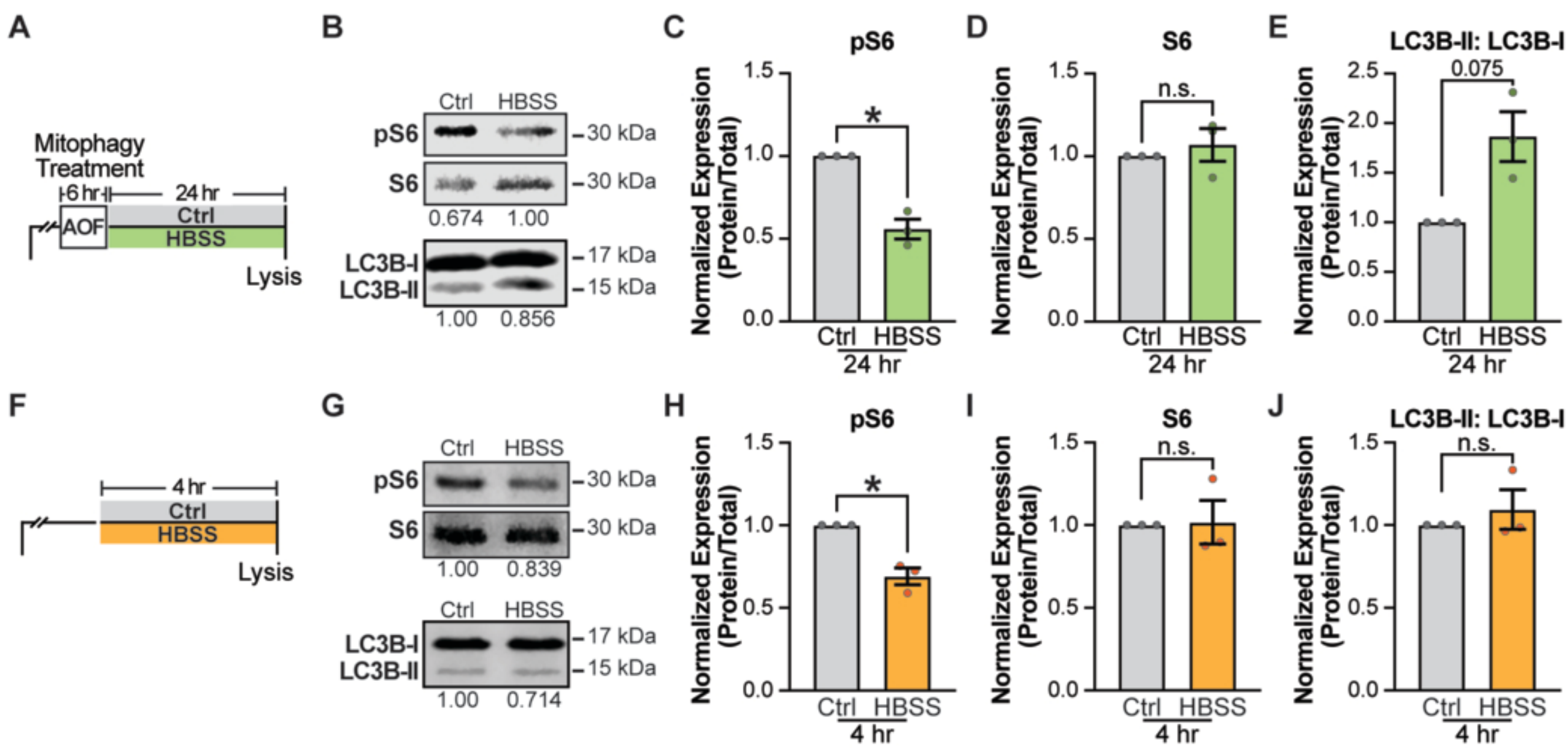

bioRxiv preprint doi: https://doi.org/10.64898/2026.01.16.700014; this version posted October 3, 2026. The copyright holder for this preprint (which was not certified by peer review) is the author/funder, who has granted bioRxiv a license to display the preprint in perpetuity. It is made available under a CC-BY 4.0 International license.

Figure S4: LC3B lipidation is enhanced after 24 hours, but not 4 hours, of partial nutrient deprivation. (**A**) Schematic illustrating the neuronal starvation experimental timeline. Briefly, a 6-hour AOF treatment induces OPTN-mediated mitophagy, followed by 24 hours in either control or HBSS to induce partial nutrient deprivation. (**B-E**) Representative fluorescent western blot (B) and quantification of pS6 (C), S6 (D), and LC3B-II:LC3B-I ratio (E) in neuron lysates. Data shown as the fold change over control of the protein of interest divided by the total protein stain. Normalization factors are shown under the corresponding representative images. Mean ± SEM; *n* = 3 biological replicates; 8-9 DIV. n.s., not significant; *, $p < 0.05$; $p = .0747$ by Welch's t-tests. (**F**) Schematic of the short partial nutrient deprivation timeline, where neurons were treated for 4 hours with either control or HBSS. (**G-J**) Representative fluorescent western blot (G) and quantification of pS6 (H), S6 (I), and LC3B-II:LC3B-I ratio (J) in neuron lysates. Data shown as the fold change over control of the protein of interest divided by the total protein stain. Normalization factors are shown under the corresponding representative images. Mean ± SEM; *n* = 3 biological replicates; 8 DIV. n.s., not significant; *, $p < 0.05$ by Welch's t-tests.

bioRxiv preprint doi: https://doi.org/10.64898/2026.01.16.700014; this version posted October 3, 2026. The copyright holder for this preprint (which was not certified by peer review) is the author/funder, who has granted bioRxiv a license to display the preprint in perpetuity. It is made available under a CC-BY 4.0 International license.

FIGURE S5

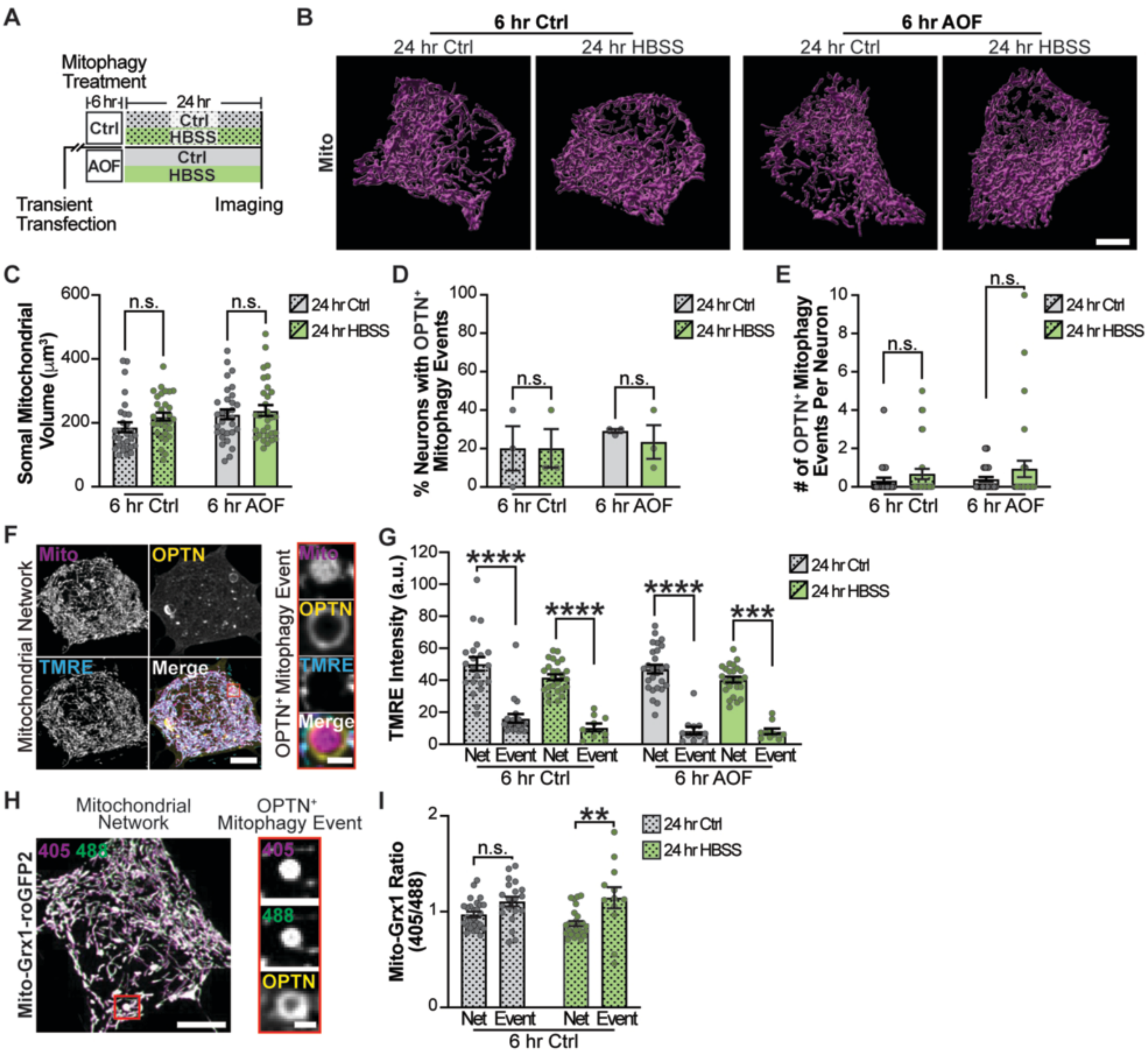

bioRxiv preprint doi: https://doi.org/10.64898/2026.01.16.700014; this version posted October 3, 2026. The copyright holder for this preprint (which was not certified by peer review) is the author/funder, who has granted bioRxiv a license to display the preprint in perpetuity. It is made available under a CC-BY 4.0 International license.

Figure S5: Partial nutrient deprivation does not affect the mitochondrial network or mitophagy induction. (**A**) Schematic illustrating a longer partial nutrient deprivation timeline. Neurons were treated for 6 hours with control or AOF, followed by 24 hours in either control or HBSS. (**B-C**) Representative images (B) and quantification (C) of somal mitochondrial surface volumes. Mean ± SEM; *n* = 29-31 neurons from 3 biological replicates; 8-9 DIV. n.s., not significant by Two-Way ANOVA with Šídák's multiple comparisons test. (**D**) Quantification of the percent of neurons undergoing OPTN-mediated mitophagy. Mean ± SEM; *n* = 9-28 events in 29-31 neurons from 3 biological replicates; 8-9 DIV. n.s., not significant by Two-Way ANOVA with Šídák's multiple comparisons test. (**E**) Quantification of the number of OPTN+ mitophagy events per neuron. Mean ± SEM; *n* = 9-28 events in 29-31 neurons from 3 biological replicates; 8-9 DIV. n.s., not significant by Two-Way ANOVA with Šídák's multiple comparisons test. (**F-G**) Representative images (F) and quantification (G) of TMRE in the mitochondrial network and at OPTN+ mitophagy events. Mean ± SEM; *n* = 9-20 events in 21-27 neurons from 3 biological replicates; 9-10 DIV. *** $p < 0.001$; **** $p < 0.0001$ by Kruskal-Wallis test with Dunn's multiple comparison test. (**H-I**) Representative images (H) and quantification (I) of Mito-Grx1-roGFP2 within the mitochondrial network and at OPTN+ mitophagy events. Scale bar, 5 μm (Left); 1 μm (Right). Mean ± SEM; *n* = 13-20 events from 3 plates per condition, which includes 27-28 neurons across 3 biological replicates; 9 DIV. n.s., not significant; **, $p < 0.01$ by Two-Way ANOVA with Tukey's multiple comparisons test. Net, network.
